# Supplementary material for: Causal Relationship between Aging and Anorexia Nervosa: A White-Matter-Microstructure-Mediated Mendelian Randomization Analysis
Source: Biomedicines. 2024 Aug 16;12(8):1874. doi: 10.3390/biomedicines12081874 (PMC11351342; doi:10.3390/biomedicines12081874)
Supplement: Supplementary file 1 [file biomedicines-12-01874-s001.zip › Supplementary Figure/Figure S4/Singe SNP plot of MR analysis for TL to ubm-b-1627.pdf]

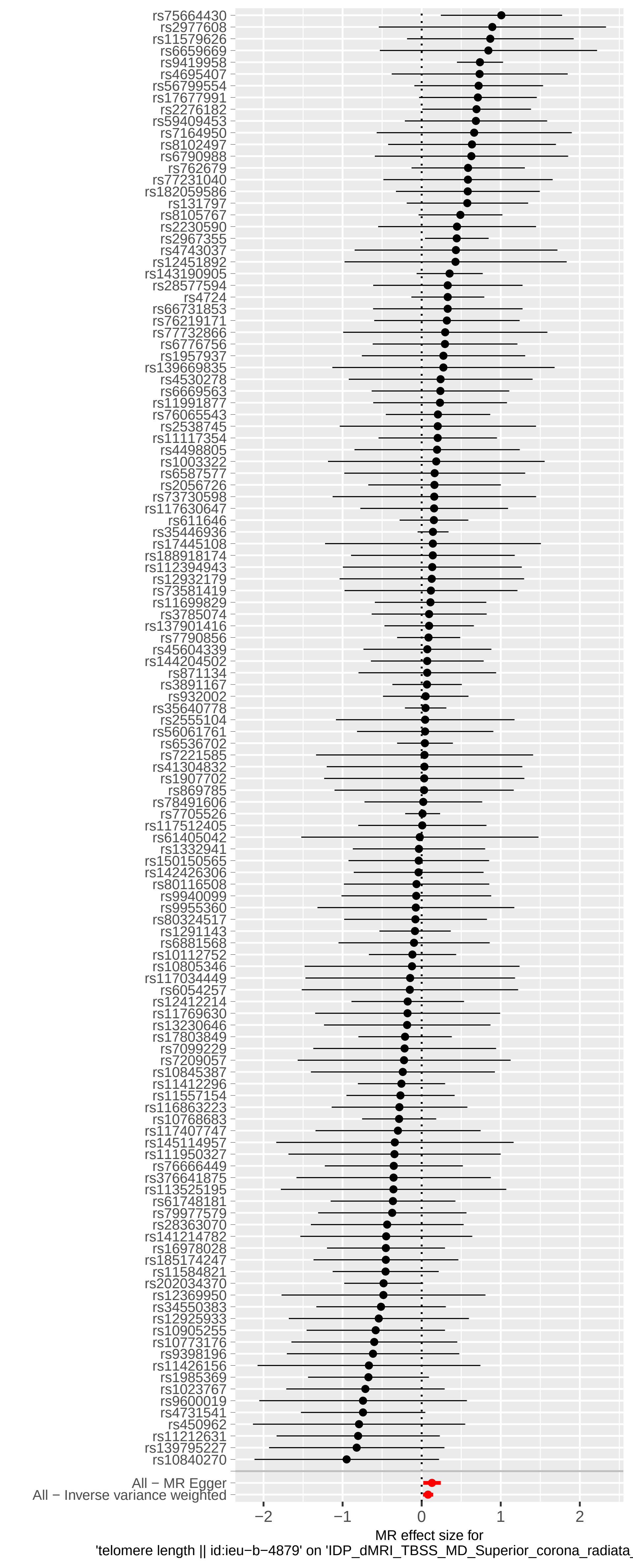

rs75664430
rs2977608
rs11579626
rs6659669
rs9419958
rs4695407
rs56799554
rs17677991
rs2276182
rs59409453
rs7164950
rs8102497
rs6790988
rs762679
rs77231040
rs182059586
rs131797
rs8105767
rs2230590
rs2967355
rs4743037
rs12451892
rs143190905
rs28577594
rs4724
rs66731853
rs76219171
rs77732866
rs6776756
rs1957937
rs139669835
rs4530278
rs6669563
rs11991877
rs76065543
rs2538745
rs11117354
rs4498805
rs1003322
rs6587577
rs2056726
rs73730598
rs117630647
rs611646
rs35446936
rs17445108
rs188918174
rs112394943
rs12932179
rs73581419
rs11699829
rs3785074
rs137901416
rs7790856
rs45604339
rs144204502
rs871134
rs3891167
rs932002
rs35640778
rs2555104
rs56061761
rs6536702
rs7221585
rs41304832
rs1907702
rs869785
rs78491606
rs7705526
rs117512405
rs61405042
rs1332941
rs150150565
rs142426306
rs80116508
rs9940099
rs9955360
rs80324517
rs1291143
rs6881568
rs10112752
rs10805346
rs117034449
rs6054257
rs12412214
rs11769630
rs13230646
rs17803849
rs7099229
rs7209057
rs10845387
rs11412296
rs11557154
rs116863223
rs10768683
rs117407747
rs145114957
rs111950327
rs76666449
rs376641875
rs113525195
rs61748181
rs79977579
rs28363070
rs141214782
rs16978028
rs185174247
rs11584821
rs202034370
rs12369950
rs34550383
rs12925933
rs10905255
rs10773176
rs9398196
rs11426156
rs1985369
rs1023767
rs9600019
rs4731541
rs450962
rs11212631
rs139795227
rs10840270
All − MR Egger
All − Inverse variance weighted
−2
−1
0
1
2
MR effect size for
'telomere length || id:ieu−b−4879' on 'IDP_dMRI_TBSS_MD_Superior_corona_radiata_
